# Mal de Mayo

**DOI:** 10.3201/eid1302.060902

**Published:** 2007-02

**Authors:** Raymond T. Foster

**Affiliations:** * Duke University Medical Center, Durham, North Carolina, USA; † 320th Combat Support Hospital Greensboro, North Carolina, USA

**Keywords:** Diarrhea, El Salvador, Mal de Mayo, another dimension

A few miles from the Honduran border, we waited for the closing ceremonies to mark the conclusion of our humanitarian efforts. Despite the oppressive, unrelenting heat, stifling body odor, the poverty surrounding us, and our longing to be reunited with family, the soldiers and airmen of Combined Joint Task Force Bravo were extremely proud of their accomplishments after months of being in El Salvador. This task force had been deployed to El Salvador as part of Operation New Horizons 2006, which is undertaken every year at multiple locations in Central and South America. As an Army physician, I had been assigned to be the task force surgeon.

The mountains of the northern Morazán region had been devastated by 14 years of civil war. But thanks to our task force, at least in part, this devastation was now softened with new schools and clinics in some of the most inaccessible areas of this nation. The backdrop for today’s celebration was a newly christened clinic devoted to gynecologic and maternal health. It seems I had left my comfortable professional environment at Duke University and traveled 1,700 miles to help construct clinics similar to those to which my civilian career was dedicated.

After the US ambassador’s remarks, I would enjoy attempts to communicate with some of the local physicians and nurses who had attended the ceremony. Despite the language barrier, we were able to communicate at a level that superseded our mutual difficulties. The happiness and hope of these professionals, of the local families present, and of the scores of uniformed school children who had been escorted there for the festivities validated our hardship and sacrifice. My thoughts turned back to the past several weeks.

I had arrived on May 17, 2006, coincident with the beginning of Central America’s rainy season. The ride from San Salvador to Camp Morazán took 3-1/2 hours and included an afternoon torrential rainstorm followed quickly by temperatures in excess of 110°F. Our engine overheated, and we were briefly stranded alongside the only hard surface road in that region.

Upon arrival at our base camp, I reported to the Sergeants Major on duty and quickly discovered that urogynecology is not the preferred medical specialty for construction accidents and tropical illness. Despite the uncomfortable conversation, I wanted the encounter to last indefinitely because the portable military air-conditioning unit attached to the headquarters tent made my temporary surroundings a pleasant, almost chilly, 90ºF.

I found the hospital tent in the dark by following the sound of the generators powering lights and essential medical equipment inside the small network of tents that formed an impromptu hospital in an El Salvadorian cow pasture. I quickly regretted that the new 80-hour work-week restrictions for my residents at Duke do not apply to Army physicians.

**Figure 1:**
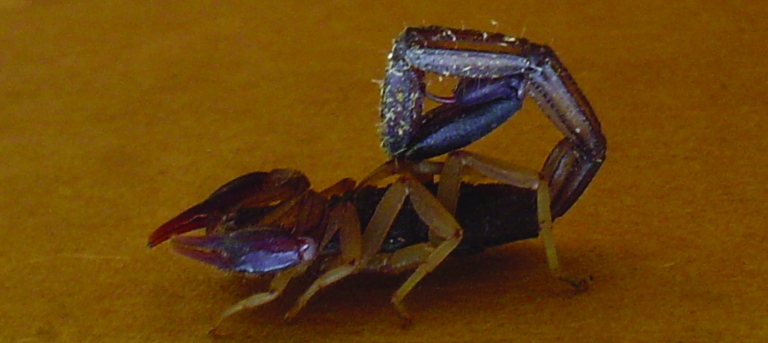
Scorpion found within the base camp hospital. Photo by R.T. Foster, Sr.

**Figure 2:**
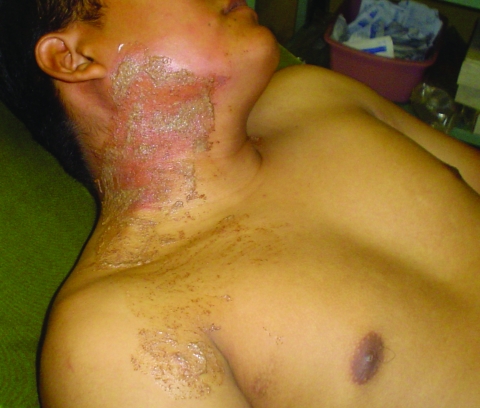
El Salvadorian infantry soldier (commando) with the characteristic rash seen after an encounter with the “pissing beetle” while sleeping.

During my first evening in the camp hospital tent, I attended to patients with minor trauma, scorpion stings (Figure 1), spider bites, and the work of the insect known affectionately as the “pissing beetle.” This insect, which I thankfully never personally experienced, was drawn to the neck and face of sleeping soldiers. A secreted liquid left an irritating and unattractive rash (Figure 2). The local remedy, juice squeezed from freshly cut limes directly onto the skin, was quite effective, and it left the otherwise pungent patients with a fresh citrus scent.

Adjacent to our base camp, a small, unkempt dwelling without electricity or running water housed a half-dozen adults and children. They shared their sparse dwelling with scores of chickens and several roosters. The fowl quickly had learned how to move through the razor wire that lined the perimeter of our camp. Thanks to these wonderful animals, I never missed an opportunity to awaken at 4:30 am, which left me ample time to prepare for breakfast in the mess tent at 5:30 am each day.

After breakfast on my first full day, I attended to patients at morning “sick call” and resumed the task of familiarizing myself with our pharmacy supplies, emergency medical equipment, evacuation procedures, and our medical documentation and charting process. At that time, it appeared as though the Army had well prepared me for various scenarios that might jeopardize the health of the more than 500 American and Salvadoran soldiers whose care was my responsibility. However, my optimism would shortly crumble.

On my third day in country, Combined Joint Task Force Bravo, which had been operating for nearly 5 months, began planning and preparation for redeployment of more than 250 military transport and heavy construction vehicles to the seaport. This meant that my hospital facilities would be disassembled and packed into shipping containers over the next 48 hours. I was to practice “tailgate medicine” from the back of 2 army ambulances, which would not be sent on the 5-hour journey to the seaport until the last convoy. I selected a small supply of medicines that I imagined would be useful for my impromptu clinic. With the ever-present stifling heat, I moved many cases of intravenous fluid into the ambulances to use for the steady flow of daily heat casualties. I armed my wheeled clinic with only 2 small bottles of antidiarrheal tablets (loperamide) and a single container of antiemetic medication (metoclopramide). I had not yet seen any gastrointestinal illness and did not anticipate that it would become a problem.

The heat, interspersed with impressive thunderstorms, continued. The day that the shipping containers packed with my mobile hospital departed for the seaport on flatbed trucks, I a saw small number of patients with acute, severe gastrointestinal symptoms. These soldiers consistently complained of copious, nearly explosive diarrhea. Other symptoms included myalgia, headache, fever, nausea, and vomiting. The frequent diarrhea, described by most soldiers as 10 or more bowel movements per day, led to dehydration for most, which was compounded by the fluid loss from the extreme heat.

To further exacerbate everyone’s misery, the plastic, portable latrines stationed around our base camp often reached interior temperatures in excess of 120ºF. Some soldiers were spending up to 2 hours per day sitting in an oven, perched above gallons of foul-smelling diarrhea. As illness grew throughout the task force, I met with our task force commander, Colonel William Buckler.

The commander already knew that key members of his staff were operating at reduced efficiency. He asked me to assess the problem so that any resources we needed from our host nation could be requested. Without medical documentation, I could only estimate that nearly half of our task force had symptomatic illness, some with severe symptoms and dehydration. Colonel Buckler became concerned not only for the welfare of soldiers under his command, both US forces and Salvadoran military personnel, but also for this disease’s effect on our ability to finish construction at remote sites near the Honduran border.

The task force staff included an Army counterintelligence agent, whom we’ll call Mr. Barreda. The mission of Mr. Barreda was to assimilate himself into the local population and notice any change in the political or social situation that might endanger our command. I was given the opportunity to meet with this crafty individual and assign him specific questions for which to seek answers during his normal interactions with the locals. Our greatest threat, however, was now identified as liquid, brown, malodorous, and ever-present in more copious amounts.

Meanwhile, assuming that patients had viral gastroenteritis, I instituted strict handwashing at meal time and in the latrine areas. I met with our cheerful yet completely overworked cook, Staff Sergeant Howard. With the help of locally hired labor, he supervised preparation of 2 hot meals, which were usually the pinnacle of our day. Sergeant Howard queried each of the food service workers about any gastrointestinal symptoms. With the assistance of a bilingual member of our command, he completed these interviews in short order. The workers reported no symptoms; however, because these local laborers feared loss of income should they be known to have a communicable disease, they may not have been completely forthcoming.

My stomach, as well as my brain, was churning as I continued to struggle with this gastrointestinal puzzle. My suspicions of a common gastroenteritis virus (such as Norwalk virus) were lessened as I noted a persistence of symptoms, especially among those with the most severe symptoms, which lingered from days to weeks. Some patients even remained ill for more than a month. My symptoms waxed and waned, consistent with constant exposure and reinfection. A confounding variable was the gastrointestinal symptoms of soldiers who were taking weekly malaria prophylaxis, which often caused similar, although less severe, digestive symptoms. In fact, many patients had already had symptoms, coincident with beginning chloroquine therapy, before even arriving in Central America.

Without access to any diagnostic aids, such as simple blood chemistries, microbiologic studies, or imaging, I might never know the exact cause of this now-rampant illness. To assess the effect on our combat strength, I instituted a diarrhea checkpoint in the chow line. Army medics asked every soldier in his or her own language, while the soldier waited to enter the food service line, if he or she had symptoms of diarrhea, nausea, or vomiting. In addition to our newly established checkpoint, I used my flashlight for daily latrine inspections. Timing inspections just before the arrival of the sanitation trucks that emptied the latrines, I was able to get a sense of how much formed stool versus liquid stool was being passed by our soldiers in each 24-hour period.

Most unexpectedly, Mr. Barreda reappeared. We had a brief conversation, and he handed me some handwritten notes, which introduced me to the phrase “mal de Mayo” (strictly translated “bad of May” and more commonly used to mean “illness of May”). Mal de Mayo was an annual event that included symptoms consistent with those of our soldiers. Mal de Mayo was associated with the rainy season, which brought countless varieties of flying insects that were presumed vectors for this poorly characterized disease. The local population was not overly concerned beyond seeking parenteral rehydration for the very young, very old, and the most severely affected. Mr. Barreda also handed me a ragged piece of paper with a short list of local remedies (in Spanish) for mal de Mayo: “cloranfenicol, yodoclorina, alka AD, intestonomicina, oreganito.”

Beyond mal de Mayo, Mr. Barreda informed me that several years earlier, Central America had experienced a rotavirus epidemic, which had caused unprecedented illness and death. Since the outbreak, the fragile El Salvadorian medical infrastructure was overwhelmed each May as patients sought reassurance that family members would not succumb to a potentially deadly infection.

My gynecologic training had not prepared me for rotavirus, other than it was a viral bowel infection particularly worrisome in newborns. I had vague recollections of *Giardia* and *Cryptosporidia*, but I was not able to recall any specific signs and symptoms beyond generic gastrointestinal complaints. My diarrhea checkpoint and daily latrine inspections led me to believe that about 60% of our forces had symptomatic illness.

Soldiers from both countries left the base camp each morning in route to construction sites. Other soldiers continued to work toward preparing and moving equipment to the port.

As an avid reader of military history, I thought often of the similarities between our mission in El Salvador and how horrific it must have been to construct the Panama Canal while battling yellow fever. History recorded that in 1884, Ferdinand de Lesseps took 500 young French engineers to Panama to supervise the construction project that he predicted would last 3 years. None of these 500 professionals lived to receive their first month’s pay. Despite this catastrophic setback, de Lesseps persisted until he lost more than 30% of his workforce consisting of 20,000 Europeans (*1*). In my estimation, Colonel William C. Gorgas (for whom the US Army hospital in Panama City is named) is the true reason the canal exists. His understanding of disease transmission, mostly gathered from the work of Major Walter Reed, led him to aggressively rid the canal construction areas of the *Aedes aegypti* mosquito (*2*). His efforts almost eliminated yellow fever from the workforce and fostered completion of the canal in 1904.

Unlike Colonel Gorgas’ success with illness plaguing the Panama Canal construction, I had no idea what might be causing disease in our population. Furthermore, aggressive field hygiene techniques, including fog treatments to rid our base camp of mosquitoes and other flying potential disease vectors, had failed to lessen the illness. All we could do was continue providing supportive care to affected soldiers. Loperamide did not usually help, and metoclopramide often increased the severity of the symptoms. Intravenous fluids and antipyretics were the mainstay of therapy. We did our best to protect ill soldiers from the heat and allow them as much rest as possible. We balanced the need for patient convalescence with our mandate for mission completion. We survived day to day, and we were all inspired by the selfless attitudes displayed by our patients. Days and weeks passed, and despite the overwhelming disease burden, the mission neared completion and task force staff shifted efforts to the planning and execution of closing ceremonies.

Within 2 days of the closing ceremonies, the base camp was disassembled beyond the point of meeting minimal requirements for soldier support. I remained in San Miguel with 4 medics and a single ambulance. In the hotel, we typically saw patients morning and night in their rooms, in effect conducting patient rounds. We continued to concentrate on supportive care, still unaware of the exact cause of the illness affecting so many members of our unit.

Twenty-two days after arriving in El Salvador, I boarded my return flight as one of the last members of the command to depart the theater of operations. During my brief tenure as task force surgeon, I became a better doctor. I never determined what causes mal de Mayo. I cannot even imagine why it spread so rapidly and affected so many people. I did, however, refocus myself on basic skills of medical practice. I took detailed histories and did countless physical exams, upon which my treatment decisions were made. I had no expensive tests to guide my decisions. I often focused much effort on simply comforting those whose symptoms were unrelenting.

At the writing of this story, I am unaware that any family members or cohabitants of soldiers who arrived home with persistent symptoms have become ill. My curiosity led me to ask questions and search the medical literature upon return from Central America. I was quickly dissatisfied with what I was able to learn from Medline investigation, internet searches, and informal conversations with colleagues trained in infectious disease. According to the Cultural Profiles Project, funded by Citizenship and Immigration Canada, Central America’s rainy season forces much of Central America’s improperly managed sewage into the water supply, leading to “dysentery and diarrhea” (*3*). I also found evidence to suggest that *Cyclospora* might be the responsible organism in contaminated drinking water in Central America (*4*). However, our soldiers were forbidden to drink from the local water supply. Bottled water and water purified by a military reverse osmosis water purification unit, tested and approved by the US Army preventive medicine team attached to the task force, was consistently available at the base camp and all construction sites.

The challenge of future US humanitarian missions in Central America will be to anticipate and care for persons affected by mal de Mayo. I am encouraged that although this disease affected the gastrointestinal tract, it missed the heart of the soldiers and airmen assigned to Combined Joint Task Force Bravo.
